# Development and validation of a clinical prediction model for diagnosing nephrotic syndrome in adults: a retrospective diagnostic prediction study

**DOI:** 10.3389/fmed.2026.1825840

**Published:** 2026-10-21

**Authors:** Xiaoyang Dong, Jing Nie, Yi Yan

**Affiliations:** Department of Dermatology, The First Affiliated Hospital of Xinjiang Medical University, Urumqi, Xinjiang, China

**Keywords:** 24-hour urinary protein excretion, adults, diagnostic prediction study, nephrotic syndrome, scoring system

## Abstract

**Background:**

The high incidence of nephrotic syndrome (NS) in adults imposes a heavy burden on families and public health care systems, highlighting the significance of early and accurate diagnosis.

**Objectives:**

To develop and internally validate a predictive model and scoring system for diagnosing NS in adults.

**Methods:**

This retrospective diagnostic prediction study enrolled 8,950 hospitalized adults with a preliminary diagnosis of NS and evidence of proteinuria from the First Affiliated Hospital of Xinjiang Medical University. The dataset was randomly divided into a training set and an internal validation set at a 7:3 ratio. Missing data were imputed separately within each set. LASSO regression was used for variable selection in the training set. Variables with univariate *P* < 0.05 were entered into a multivariable logistic model using bidirectional stepwise selection. Model discrimination, calibration, and clinical utility were evaluated. Internal validation was performed using the bootstrap-impute-predict framework with 500 bootstrap samples. A diagnostic scoring system and risk stratification were subsequently developed.

**Results:**

LASSO regression retained 10 key variables, and the final multivariable logistic model included 9 variables: age, hypertension, hyperlipidemia, edema, 24-hour urinary protein excretion, urine protein qualitative result, serum albumin, cystatin C, and D-dimer. The likelihood ratio tests were statistically significant in both the training and internal validation sets (both *P* < 0.001). The apparent AUC was 0.89 (95% CI: 0.89–0.90) in the training set, and the Harrell-corrected AUC remained 0.89 after 500 bootstrap resamples. The AUC was 0.90 (95% CI: 0.89–0.91) in the internal validation set. Calibration curves indicated good agreement, and decision curve analysis demonstrated clinical net benefit. Sensitivity analyses further supported the robustness of the model. The score-based system effectively distinguished patients with different risks of NS, and the observed proportion of patients with NS increased progressively with higher scores.

**Conclusions:**

In hospitalized adults with suspected NS and evidence of proteinuria, this model and scoring system may support early diagnostic assessment and risk stratification, pending external validation.

## Introduction

Nephrotic syndrome (NS) results from injury to the glomerular filtration barrier and is characterized by nephrotic-range proteinuria, hypoalbuminemia, edema, and hyperlipidemia. Primary causes include minimal change disease, membranous nephropathy, and focal segmental glomerulosclerosis. Secondary causes include diabetes mellitus, systemic lupus erythematosus, and amyloidosis (1, 2). Contemporary epidemiological data indicate that the overall incidence of NS is 3.85 per 100,000 person-years, with a sustained upward trend observed over the past two decades (3). Notably, the incidence is marginally higher in males than in females (3). Beyond its considerable disease burden, NS is associated with substantial mortality risk: the 30-day mortality rate reaches 4.6%, with cumulative 1-year and 5-year mortality rates of 14% and 33%, respectively (3). All-cause mortality is 5.45 times higher in patients with NS than in the general population (4). Additionally, patients with NS face substantial risks of severe complications, including acute kidney injury (AKI) (5) and a 2.56-fold increased risk of venous thromboembolism (6, 7). Some patients develop NS at a younger age and therefore experience longer disease duration and cumulative complication burdens, underscoring the importance of early recognition and accurate diagnosis.

Currently, 24-hour urinary protein excretion (24 h UPE), a core diagnostic marker for NS, is associated with suboptimal patient compliance and cumbersome specimen collection procedures (8). Critically, NS often presents with atypical clinical manifestations, with thromboembolic complications or AKI as the initial presenting features (5, 9), which frequently leads to delays in diagnosis and treatment. Although renal biopsy is essential for determining the underlying glomerular pathology, it is invasive, costly, subject to contraindications, and dependent on specialized pathology resources and pathologists. It is therefore unsuitable for population-based screening or initial triage of patients with suspected NS (10).

Limited diagnostic capacity and clinical experience in primary care settings may contribute to delayed or incorrect diagnosis, potentially delaying optimal treatment. Delayed diagnosis and intervention can lead to severe clinical complications, including AKI, deep vein thrombosis, and severe infections. Notably, some patients already present with rapidly progressive renal deterioration at the time of overt clinical manifestation of NS (5, 6, 11). Consequently, the development of diagnostic models based on routine clinical and laboratory parameters is urgently warranted for the early and rapid identification of NS. Such models may be useful in hospital settings to support the evaluation and triage of patients with suspected NS after proteinuria has been detected. Their performance in outpatient or primary-care settings remains to be determined.

With the continuous development of clinical epidemiology and statistical methods, prediction models based on multi-factor integration have become an important tool to improve the accuracy of disease diagnosis. By combining clinical symptoms with laboratory results, these models estimate individual disease risk and provide an objective basis for diagnosis (12). However, current studies on prediction models specifically designed for the early diagnosis of NS remain scarce. Existing models have mainly focused on pathological classification or prognosis and are often limited by small sample sizes and suboptimal clinical interpretability (13, 14). Therefore, this study aimed to develop and internally validate a readily interpretable NS diagnostic prediction model using large-sample clinical data from our hospital. By integrating a risk scoring system, this model may serve as a diagnostic-assist tool for clinicians in identifying and stratifying hospitalized patients with suspected NS and evidence of proteinuria.

## Methods

### Study design and study participants

This was a retrospective diagnostic prediction study. Patients with a preliminary diagnosis of NS admitted to the First Affiliated Hospital of Xinjiang Medical University between January 2012 and July 2025 were screened, and 8,950 eligible patients were enrolled after the inclusion and exclusion criteria were applied. The inclusion criteria were as follows: 1) Age ≥ 18 years; 2) Meeting any one of the following clinical indicators: a) 24 h UPE ≥ 3.5 g/24 h, with or without edema; b) random urine protein qualitative test ≥ 3+, with or without edema. The exclusion criteria included: 1) Secondary NS caused by any underlying diseases, including but not limited to systemic lupus erythematosus-related nephritis, diabetic nephropathy, ANCA-associated vasculitis-related renal injury, Sjögren syndrome-related renal injury, rheumatoid arthritis-related renal injury, Henoch-Schönlein purpura nephritis, myeloma-related nephropathy, and other secondary causes; 2) A previous definitive diagnosis of NS before admission; 3) Concurrent severe hepatic and renal failure; 4) Severe deficiency of clinical data; 5) Pregnant females. This study was approved by the Ethics Committee of the First Affiliated Hospital of Xinjiang Medical University (Ethics Approval No: K202509-59) and conducted in strict accordance with the ethical principles of the World Medical Association Declaration of Helsinki, as well as national medical research ethical guidelines. Due to its retrospective design, all data were extracted from routine clinical records without additional interventions, and patient personal information was fully anonymized to protect privacy; a waiver of informed consent was granted by the ethics committee following review.

### Data collection

We collected baseline data and the initial laboratory results obtained on admission from all hospitalized patients with a preliminary diagnosis of NS. The variables were grouped as follows. (1) Demographic characteristics: age and sex. (2) History and physical findings: smoking, alcohol use, hypertension, hyperlipidemia, and edema. (3) Laboratory findings: 24 h UPE, urine protein qualitative result, serum albumin, serum creatinine, uric acid, urea, cystatin C, potassium, sodium, magnesium, D-dimer, procalcitonin, and interleukin-6. (4) Outcome: diagnosis of NS (yes/no).

### Missing data processing

Variables with a missing rate exceeding 20% were excluded from further analysis. Before imputation, patients had already been assigned to the training and internal validation sets, as described below. Missing values were then imputed using deterministic regression imputation with fully observed covariates as predictors, performed separately within the training and internal validation sets, following the bootstrap-impute-predict framework proposed by Mi et al. (15). No imputation model included the outcome. To simultaneously account for imputation-related uncertainty and model overfitting, an optimism-corrected estimate of model performance was obtained within the training set. Imputation quality was further evaluated by comparing the distributions (means and standard deviations) of observed and imputed values for each variable with missing data.

### Study outcome events

The primary outcome of this study was the status of definitive diagnosis of NS at discharge in patients who had a preliminary diagnosis of NS on admission. The diagnosis of NS was based on the Kidney Disease: Improving Global Outcomes (KDIGO) 2021 Clinical Practice Guideline for Glomerular Diseases (16), which clearly defines NS by the presence of two mandatory criteria: 24 h UPE ≥ 3.5 g and serum albumin <30 g/L, with edema and hyperlipidemia as common associated clinical manifestations rather than mandatory diagnostic criteria. For patients with high clinical suspicion of NS but whose clinical data did not meet the above KDIGO diagnostic criteria (i.e., uncertain clinical diagnosis), renal biopsy was performed. Patients whose histopathological results were consistent with the characteristic glomerular lesions of NS were also diagnosed as having NS and included in the NS group.

### Statistical analysis

Statistical analyses were performed using R version 4.5.2 (R Foundation for Statistical Computing, Vienna, Austria), and the R packages used included tidyverse [2.0.0], dplyr [1.1.4], tidyr [1.3.1], ggplot2 [3.5.1], glmnet [4.1.7], car [3.1.2], broom [1.0.5], pROC [1.18.0], caret [6.0.94], rmda [1.6.0], rms [6.7.0], calibrationCurves [2.0.0] and MASS [7.3–65]. Continuous variables with a skewed distribution were described as median (interquartile range), and the Wilcoxon-Mann–Whitney test was used for intergroup comparisons. Categorical variables were presented as number (percentage) [n(%)], and intergroup comparisons were conducted using the chi-square test or Fisher's exact test.

The dataset was randomly divided into a training set and an internal validation set at a 7:3 ratio, stratified by the final diagnosis of NS (random seed = 7) to ensure comparable distributions of NS and non-NS patients between the two sets. After missing data were imputed separately within each set, LASSO regression was applied to the training set for variable selection, in which the L1 penalty term was embedded into the objective function and the lambda parameter was optimized by ten-fold cross-validation; variables with non-zero coefficients at lambda.1se were retained as candidate variables and were then categorized according to predefined clinical reference ranges for subsequent logistic modeling. Multicollinearity among the retained variables was evaluated using the Generalized Variance Inflation Factor (GVIF); variables with GVIF^(1/(2  ×  Df)) > 5 were considered to have significant multicollinearity and were excluded.

Univariate logistic regression analysis was performed in the training set, and variables with *P* < 0.05 were entered into the multivariable logistic regression model using bidirectional stepwise regression. The discriminative ability of the model was quantified using the area under the receiver operating characteristic (ROC) curve (AUC) with 95% confidence interval (CI); the optimal cutoff was determined by the Youden index, and accuracy, sensitivity, specificity, positive predictive value (PPV), and negative predictive value (NPV) were calculated. The AUCs of the training and internal validation sets were compared using the independent-samples DeLong's test. Model fit was assessed using the likelihood ratio test. Calibration slope, calibration intercept, and the Brier score were calculated to quantify calibration performance. A nomogram was constructed from the final multivariable model to visualize the individual probability of NS. Calibration curves and decision curve analysis (DCA) were adopted to evaluate the goodness of fit and clinical utility of the model, respectively. Because missing values were present in the original data and the model was developed within the training set, the bootstrap-impute-predict approach (15) was used for internal validation within the training set: 500 bootstrap samples were drawn with replacement, and the same deterministic imputation and model-fitting procedures were repeated within each bootstrap sample. Performance was evaluated in each bootstrap sample and in the original training data; the mean difference between the two estimates defined the optimism. Subtracting this optimism from the apparent AUC yielded the Harrell-corrected AUC, which we report as the internally validated estimate of discrimination. The internal validation set was used only for final model evaluation.

To assess whether model performance depended on proteinuria-related predictors, we performed two sensitivity analyses using the same training and validation split as the primary analysis. In the first analysis, 24 h UPE and qualitative urine protein were excluded, and the model was refitted using the remaining seven variables, with the same coding as the primary analysis. In the second analysis, serum albumin was further excluded, and the model was refitted using the remaining six variables. For each reduced model, AUC with 95% CI, accuracy, sensitivity, specificity, PPV, and NPV were calculated in the training and internal validation sets. These analyses were used to evaluate the robustness of the model and the diagnostic contribution of the remaining predictors.

Based on the results of the multivariable logistic regression, this study established a risk scoring table for NS with reference to the risk scoring function method of the Framingham study (17); in addition, graded analysis of the scoring effect was conducted to further verify the stability of the model. Two-tailed tests were used in all analyses of this study, and *P* < 0.05 was considered statistically significant.

### Sample size

All consecutive eligible patients admitted during the study period were included, and no *a priori* sample size calculation was performed. We therefore assessed whether the sample had been sufficient for model development and evaluation, using the framework of Riley et al. (18). Assuming the observed outcome prevalence (0.5084), 16 predictor parameters, and a conservative anticipated C-statistic of 0.80, the minimum sample size required for model development was approximately 470 patients (approximately 240 events). The requirement remained well below the available sample size even assuming 28 parameters (861 patients). The internal validation set (2,685 patients, 1,365 events) was likewise sufficient for evaluating model performance. The training set comprised 6,265 patients with 3,185 outcome events (approximately 199 events per predictor parameter), substantially exceeding both this minimum and the conventional threshold of 10 events per variable.

## Results

### Study population and missing data handling

A total of 15,447 adult patients with a preliminary diagnosis of NS were initially included. After applying the exclusion criteria (2,108 with secondary NS, 1,620 with severe hepatic and renal insufficiency, 2,512 with a previous definitive diagnosis of NS, 215 with severe deficiency of clinical data, and 42 pregnant females), 8,950 patients were finally enrolled (Supplementary Figure 1). After stratification by the final diagnosis of NS, patients were randomly divided into a training set (*n* = 6,265) and an internal validation set (*n* = 2,685), with an NS proportion of 50.84% in both sets.

Among the 20 candidate variables, 12 had missing values, with missing rates ranging from 0.85% to 18.27%; D-dimer had the highest missing rate (18.27%). After deterministic regression imputation was performed separately in the training and internal validation sets, observed and imputed values were compared for each variable with missing data. For most variable-dataset comparisons (87.5%), the observed and imputed values showed comparable central distributions, and the standard deviations of imputed values were generally smaller than those of observed values, which is consistent with the characteristic of deterministic regression imputation using conditional means (Supplementary Table 1).

### LASSO variable selection in the training set

LASSO regression was performed on the candidate variables in the training set (*n* = 6,265). Using the lambda.1se criterion, 10 key variables were retained, including age, hypertension, hyperlipidemia, edema, 24 h UPE, qualitative urine protein, serum creatinine, serum albumin, cystatin C, and D-dimer. The detailed selection process is presented in Supplementary Figure 2.

### Comparison of baseline characteristics between the training and validation sets

Comparisons between the training and internal validation sets showed that before variable assignment, no statistically significant differences were observed in the NS proportion, age, 24 h UPE, Scr, CysC, D-dimer, hypertension, hyperlipidemia, edema, or qualitative urine protein (all *P* > 0.05), except for a slight difference in serum albumin (median 28.70 vs. 28.00 g/L, *P* = 0.011). After variable assignment according to the prespecified clinical reference ranges, all variables were comparable between the two sets (all *P* > 0.05). These findings indicate that the training and internal validation sets were generally balanced after variable assignment and suitable for subsequent model development and validation. The variable assignment rules are shown in Supplementary Table 2, and the comparisons before and after variable assignment are shown in Supplementary Tables 3, 4, respectively.

### Comparison between NS and non-NS patients in the training set

In the training set, the NS group had significantly higher proportions of hypertension, hyperlipidemia, edema, 24 h UPE ≥3.5 g/24 h, urine protein qualitative 3 + or 4+, serum albumin <39 g/L, D-dimer ≥280 ng/mL, and cystatin C > 1.15 mg/L than the non-NS group, and it also had a higher proportion of patients aged 18 to 44 years (all *P* < 0.05). Serum creatinine categories did not differ significantly between the two groups (*P* = 0.056). The detailed results are shown in Table 1.

**Table 1:** Comparison of the NS and non-NS groups in the training set.

Variables	Total (*n* = 6,265)	non-NS (*n* = 3,080)	NS (*n* = 3,185)	*χ* ^2^	P
Age, *n* (%)				82.23	<0.001
≥65	1,306 (20.85)	757 (24.58)	549 (17.24)		
45–64	2,744 (43.80)	1,383 (44.90)	1,361 (42.73)		
18–44	2,215 (35.36)	940 (30.52)	1,275 (40.03)		
Hypertension, *n* (%)				38.84	<0.001
No	2,928 (46.74)	1,563 (50.75)	1,365 (42.86)		
Yes	3,337 (53.26)	1,517 (49.25)	1,820 (57.14)		
Hyperlipidemia, *n* (%)				254.12	<0.001
No	5,051 (80.62)	2,733 (88.73)	2,318 (72.78)		
Yes	1,214 (19.38)	347 (11.27)	867 (27.22)		
Edema, *n* (%)				1,091.37	<0.001
No	2,843 (45.38)	2,049 (66.53)	794 (24.93)		
Yes	3,422 (54.62)	1,031 (33.47)	2,391 (75.07)		
24 h UPE, *n* (%)				2,264.38	<0.001
<0.15	1,386 (22.12)	1,227 (39.84)	159 (4.99)		
0.15–3.499	2,965 (47.33)	1,707 (55.42)	1,258 (39.50)		
≥3.5	1,914 (30.55)	146 (4.74)	1,768 (55.51)		
Urine protein qualitative, *n* (%)				2,174.64	<0.001
Negative	1,248 (19.92)	1,077 (34.97)	171 (5.37)		
1+	1,097 (17.51)	889 (28.86)	208 (6.53)		
2+	1,198 (19.12)	613 (19.90)	585 (18.37)		
3+	2,595 (41.42)	493 (16.01)	2,102 (66.00)		
4+	127 (2.03)	8 (0.26)	119 (3.74)		
Serum albumin, *n* (%)				417.63	<0.001
39–54	1,179 (18.82)	880 (28.57)	299 (9.39)		
<39	5,036 (80.38)	2,156 (70.00)	2,880 (90.42)		
>54	50 (0.80)	44 (1.43)	6 (0.19)		
D-dimer, *n* (%)				488.02	<0.001
<280	3,468 (55.36)	2,140 (69.48)	1,328 (41.70)		
≥280	2,797 (44.64)	940 (30.52)	1,857 (58.30)		
Cystatin C, *n* (%)				150.04	<0.001
0.56–1.15	3,341 (53.33)	1,873 (60.81)	1,468 (46.09)		
<0.56	150 (2.39)	84 (2.73)	66 (2.07)		
>1.15	2,774 (44.28)	1,123 (36.46)	1,651 (51.84)		
Serum creatinine, *n* (%)				5.76	0.056
33–75	2,898 (46.26)	1,462 (47.47)	1,436 (45.09)		
<33	41 (0.65)	15 (0.49)	26 (0.82)		
>75	3,326 (53.09)	1,603 (52.05)	1,723 (54.10)		

χ^2^, chi-square test; 24 h UPE, 24-hour urinary protein excretion; NS, nephrotic syndrome.

### Univariate and multivariate logistic regression results of the training set

In the training set, univariate logistic regression showed that age, hypertension, hyperlipidemia, edema, 24 h UPE, urine protein qualitative, serum albumin, D-dimer, and cystatin C were associated with NS diagnosis. Serum creatinine was not statistically associated with NS diagnosis. The nine variables with univariate *P* < 0.05 were included in multivariable logistic regression. Compared with their respective reference categories, age 45 to 64 years (OR=1.74) and 18 to 44 years (OR=2.64), hypertension (OR=1.51), hyperlipidemia (OR=1.98), edema (OR=2.55), 24 h UPE 0.15 to <3.5 g/24 h (OR=2.58) and ≥3.5 g/24 h (OR=14.46), urine protein qualitative 2+ (OR=1.77), 3+ (OR=4.07), and 4+ (OR=8.06), serum albumin <39 g/L (OR=1.86), D-dimer ≥280 ng/mL (OR=1.55), and cystatin C > 1.15 mg/L (OR=1.17) were associated with an increased risk of NS diagnosis. In the multivariable model, urine protein qualitative 1+ (OR=0.81), serum albumin >54 g/L (OR=0.45), and cystatin C < 0.56 mg/L (OR=0.94) did not differ significantly from their respective reference categories. Detailed results are shown in Table 2. The GVIF values for each variable in the final model are presented in Supplementary Table 5, and all adjusted GVIF^(1/(2  ×  Df)) values were below 5, indicating no significant multicollinearity.

**Table 2:** Univariate and multivariable logistic regression in the training set.

Variables	Univariate					Multivariate				
	*β*	S.E	Z	P	OR (95%CI)	β	S.E	Z	P	OR (95%CI)
Age										
≥65					1.00 (Reference)					1.00 (Reference)
[45, 64]	0.31	0.07	4.50	<0.001	1.36 (1.19–1.55)	0.55	0.10	5.65	<0.001	1.74 (1.43–2.10)
[18, 44]	0.63	0.07	8.86	<0.001	1.87 (1.63–2.15)	0.97	0.11	8.83	<0.001	2.64 (2.13–3.27)
Hypertension										
No					1.00 (Reference)					1.00 (Reference)
Yes	0.32	0.05	6.25	<0.001	1.37 (1.24–1.52)	0.41	0.08	5.41	<0.001	1.51 (1.30–1.75)
Hyperlipidemia										
No					1.00 (Reference)					1.00 (Reference)
Yes	1.08	0.07	15.54	<0.001	2.95 (2.57–3.38)	0.68	0.09	7.21	<0.001	1.98 (1.64–2.38)
Edema										
No					1.00 (Reference)					1.00 (Reference)
Yes	1.79	0.06	31.95	<0.001	5.98 (5.36–6.68)	0.94	0.07	12.89	<0.001	2.55 (2.21–2.94)
24 h UPE										
[0, 0.15)					1.00 (Reference)					1.00 (Reference)
[0.15, 3.5)	1.74	0.09	18.87	<0.001	5.69 (4.75–6.81)	0.95	0.11	8.26	<0.001	2.58 (2.06–3.24)
≥3.5	4.54	0.12	37.66	<0.001	93.45 (73.79–118.34)	2.67	0.15	18.17	<0.001	14.46 (10.84–19.29)
Urine protein qualitative										
Negative					1.00 (Reference)					1.00 (Reference)
1+	0.39	0.11	3.44	<0.001	1.47 (1.18–1.84)	−0.20	0.13	−1.54	0.122	0.81 (0.63–1.06)
2+	1.79	0.10	17.83	<0.001	6.01 (4.94–7.32)	0.57	0.13	4.46	<0.001	1.77 (1.38–2.27)
3+	3.29	0.10	34.16	<0.001	26.85 (22.23–32.43)	1.40	0.13	11.09	<0.001	4.07 (3.18–5.22)
4+	4.54	0.37	12.13	<0.001	93.69 (44.98–195.15)	2.09	0.40	5.16	<0.001	8.06 (3.65–17.79)
Serum albumin										
[39, 54]					1.00 (Reference)					1.00 (Reference)
<39	1.37	0.07	18.82	<0.001	3.93 (3.41–4.53)	0.62	0.10	6.52	<0.001	1.86 (1.54–2.25)
>54	−0.91	0.44	−2.07	0.038	0.40 (0.17–0.95)	−0.80	0.48	−1.68	0.092	0.45 (0.18–1.14)
D-dimer										
[0, 280)					1.00 (Reference)					1.00 (Reference)
≥280	1.16	0.05	21.80	<0.001	3.18 (2.87–3.53)	0.44	0.08	5.58	<0.001	1.55 (1.33–1.80)
Cystatin C										
[0.56, 1.15]					1.00 (Reference)					1.00 (Reference)
<0.56	0.00	0.17	0.01	0.988	1.00 (0.72–1.39)	−0.06	0.23	−0.27	0.786	0.94 (0.59–1.49)
>1.15	0.63	0.05	12.08	<0.001	1.88 (1.69–2.08)	0.15	0.07	2.08	0.038	1.17 (1.01–1.35)
Serum creatinine										
[33, 75]					1.00 (Reference)					
<33	0.57	0.33	1.74	0.082	1.76 (0.93–3.35)					
>75	0.09	0.05	1.77	0.076	1.09 (0.99–1.21)					

24 h UPE, 24-hour urinary protein excretion; OR, odds ratio; CI, confidence interval.

### Evaluation and presentation of the NS diagnostic model

The final multivariable logistic regression model was developed in the training set and included nine variables (16 indicator terms), the coefficients of which are presented in Table 2. The complete prediction equation is given in Equation (1):

logit(P) = −4.02 + 0.55  ×  I(45 ≤ Age ≤ 64 years) + 0.97 × I(18 ≤ Age ≤ 44 years) + 0.41 × I(Hypertension = Yes) + 0.68 × I(Hyperlipidemia = Yes) + 0.94 × I(Edema = Yes) + 0.95 × I(0.15 ≤ 24 h UPE < 3.5 g/24 h) + 2.67 × I(24 h UPE ≥ 3.5 g/24 h)−0.20 × I(Urine protein qualitative = 1+) + 0.57 × I(Urine protein qualitative = 2+) + 1.40 × I(Urine protein qualitative = 3+) + 2.09 × I(Urine protein qualitative = 4+) + 0.62 × I(Serum albumin < 39 g/L)−0.80 × I(Serum albumin > 54 g/L) + 0.44 × I(D-dimer ≥ 280 ng/mL)−0.06 × I(Cystatin C < 0.56 mg/L) + 0.15 × I(Cystatin C > 1.15 mg/L)

where logit(P) = ln[P/(1−P)]; P denotes the predicted probability of a definitive diagnosis of nephrotic syndrome (NS) at discharge; I(·) is an indicator function that equals 1 when the corresponding condition is met and 0 otherwise; and 24 h UPE denotes 24-hour urinary protein excretion. The reference categories were age ≥65 years, no hypertension, no hyperlipidemia, no edema, 24 h UPE <0.15 g/24 h, a negative urine protein qualitative result, serum albumin 39–54 g/L, D-dimer <280 ng/mL, and cystatin C 0.56–1.15 mg/L. Individual predicted probabilities were obtained as *P* = 1/(1 + exp(−logit(P))). The variable assignment rules are shown in Supplementary Table 2.

ROC curves were generated in the training and internal validation sets to evaluate model discrimination (Figure 1A). The apparent AUC in the training set was 0.89 (95% CI 0.89–0.90). After 500 bootstrap resamples, the Harrell-corrected AUC remained 0.89, indicating limited overfitting. The AUC in the internal validation set was 0.90 (95% CI 0.89–0.91). A DeLong's test showed no significant difference in AUC between the two sets (Z = −0.830, *P* = 0.407), indicating no significant difference in model discrimination between the two sets. The nomogram based on the final multivariable logistic regression model is shown in Figure 1B. It can be used to estimate the individual probability of NS, with 24 h UPE showing a prominent contribution to model prediction. DCA showed positive net benefit across a broad range of clinically relevant threshold probabilities in both the training and internal validation sets (Figures 1C,D), supporting its potential clinical utility within this cohort. Calibration curves showed good agreement between predicted probabilities and observed outcomes (Figures 1E,F). The calibration slope and intercept were 1.00 and 0.00 in the training set, with a Brier score of 0.13. The corresponding values in the internal validation set were 1.05 and 0.02, with a Brier score of 0.12. These findings suggest that the predicted probabilities were reasonably accurate and stable within the training and internal validation sets. Model verification showed statistically significant likelihood ratio tests in both sets (training set: *χ*^2^ = 3,554.014, df = 16, *P* < 0.001, internal validation set: *χ*^2^ = 1,606.323, df = 16, *P* < 0.001) (Table 3). Classification metrics at the optimal cutoff (0.521 in both sets) were closely comparable between the two sets (Table 4).

**Figure 1:**
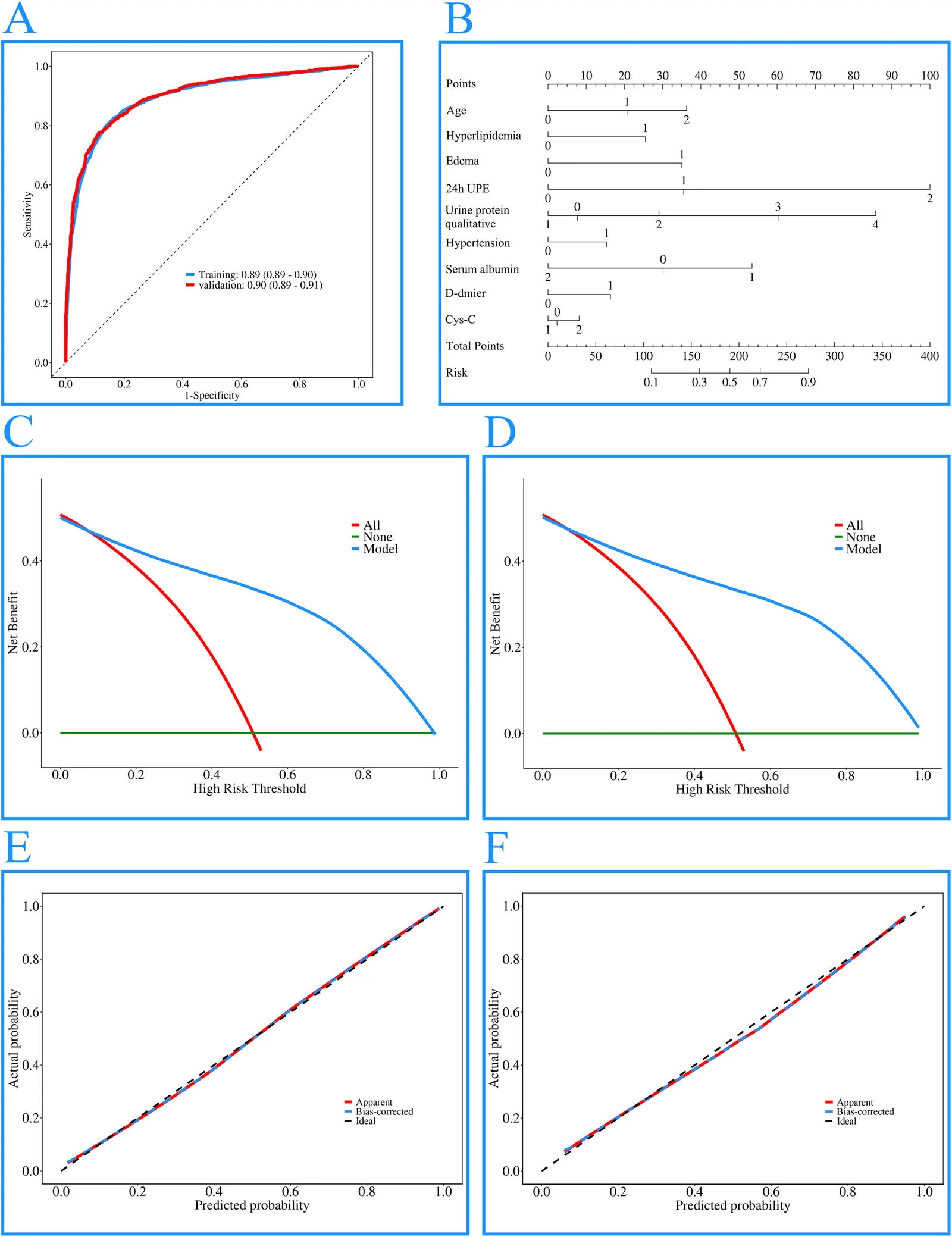
Diagnostic model performance evaluation metrics. **(A)** ROC curve of the training and internal validation sets; **(B)** Nomogram; **(C)** DCA of the training set; **(D)** DCA of the internal validation set; **(E)** Calibration curve of the training set; **(F)** Calibration curve of the internal validation set.

**Table 3:** Model verification, discrimination and calibration of the NS diagnostic model.

Evaluation direction	Evaluation content	Training set		Internal validation set	
		Statistics	P	Statistics	P
Model verification	Likelihood ratio test	χ^2^ = 3,554.014	<0.001	χ^2^ = 1,606.323	<0.001
Discrimination assessment	C-index	0.89 (0.89–0.90)	<0.001	0.90 (0.89–0.91)	<0.001
Calibration assessment	Calibration slope	1.00	-	1.05	-
Calibration assessment	Calibration intercept	0.00	-	0.02	-
Calibration assessment	Brier score	0.13	-	0.12	-

**Table 4:** Diagnostic performance of the NS diagnostic model at the optimal cut-off value.

Data	AUC (95%CI)	Accuracy (95%CI)	Sensitivity (95%CI)	Specificity (95%CI)	PPV (95%CI)	NPV (95%CI)	Cutoff
Training set	0.89 (0.89–0.90)	0.83 (0.82–0.84)	0.79 (0.78–0.81)	0.87 (0.85–0.88)	0.86 (0.85–0.87)	0.80 (0.79–0.82)	0.521
Internal validation set	0.90 (0.89–0.91)	0.83 (0.81–0.84)	0.79 (0.77–0.81)	0.86 (0.85–0.88)	0.86 (0.84–0.88)	0.80 (0.78–0.82)	0.521

AUC, area under the curve; PPV, positive predictive value; NPV, negative predictive value.

In sensitivity analyses, after excluding 24 h UPE and urine protein qualitative, the remaining seven variables remained significantly associated with NS, with AUCs of 0.81 in both the training and internal validation sets. After further excluding serum albumin, the AUCs were 0.80 in both sets, suggesting that the model had some degree of stability. Detailed metrics are shown in Supplementary Table 6, and ROC curves are presented in Supplementary Figure 3A, B.

### Development of a diagnostic scoring table for NS and clinical risk stratification assessment

Based on the Framingham scoring approach, we developed an NS diagnostic scoring table using the final multivariable logistic regression model. Points were assigned as follows: age 45 to 64 years, 4 points. Age 18 to 44 years, 6 points. Hypertension, hyperlipidemia, and edema, 3, 5, and 6 points, respectively. A 24 h UPE of 0.15 to 3.5 g/24 h, 6 points. A 24 h UPE of ≥3.5 g/24 h, 18 points. Urine protein qualitative results of 2+, 3+, and 4+, 4, 9, and 14 points, respectively. Serum albumin <39 g/L, 4 points. D-dimer ≥280 ng/mL, 3 points. Cystatin C > 1.15 mg/L, 1 point. All other levels were assigned 0 points (Table 5).

**Table 5:** Scoring table of the NS diagnostic model.

Variable	Category	Points
Age, years	[45, 64]	4
Age, years	[18, 44]	6
Hypertension	Yes	3
Hyperlipidemia	Yes	5
Edema	Yes	6
24 h UPE, g/24h	[0.15, 3.5)	6
24 h UPE, g/24h	≥3.5	18
Urine protein qualitative	2+	4
Urine protein qualitative	3+	9
Urine protein qualitative	4+	14
Serum albumin, g/L	<39	4
D-dimer, ng/mL	≥280	3
Cystatin C, mg/L	>1.15	1

24 h UPE, 24-hour urinary protein excretion; NS, nephrotic syndrome.

The tertiles of the total score in the training set were used as risk cutoffs. Patients were classified as low risk LR 0 to 19 points, moderate risk MR 20 to 35 points, and high risk HR 36 to 60 points. The same cutoffs were applied to the internal validation set. Both the training and internal validation sets showed a clear increase in the proportion of patients with NS as the total score increased (Supplementary Table 7 and Figure 2). Using the LR group as the reference, both the MR and HR groups were significantly associated with an increased risk of NS (Table 6).

**Figure 2:**
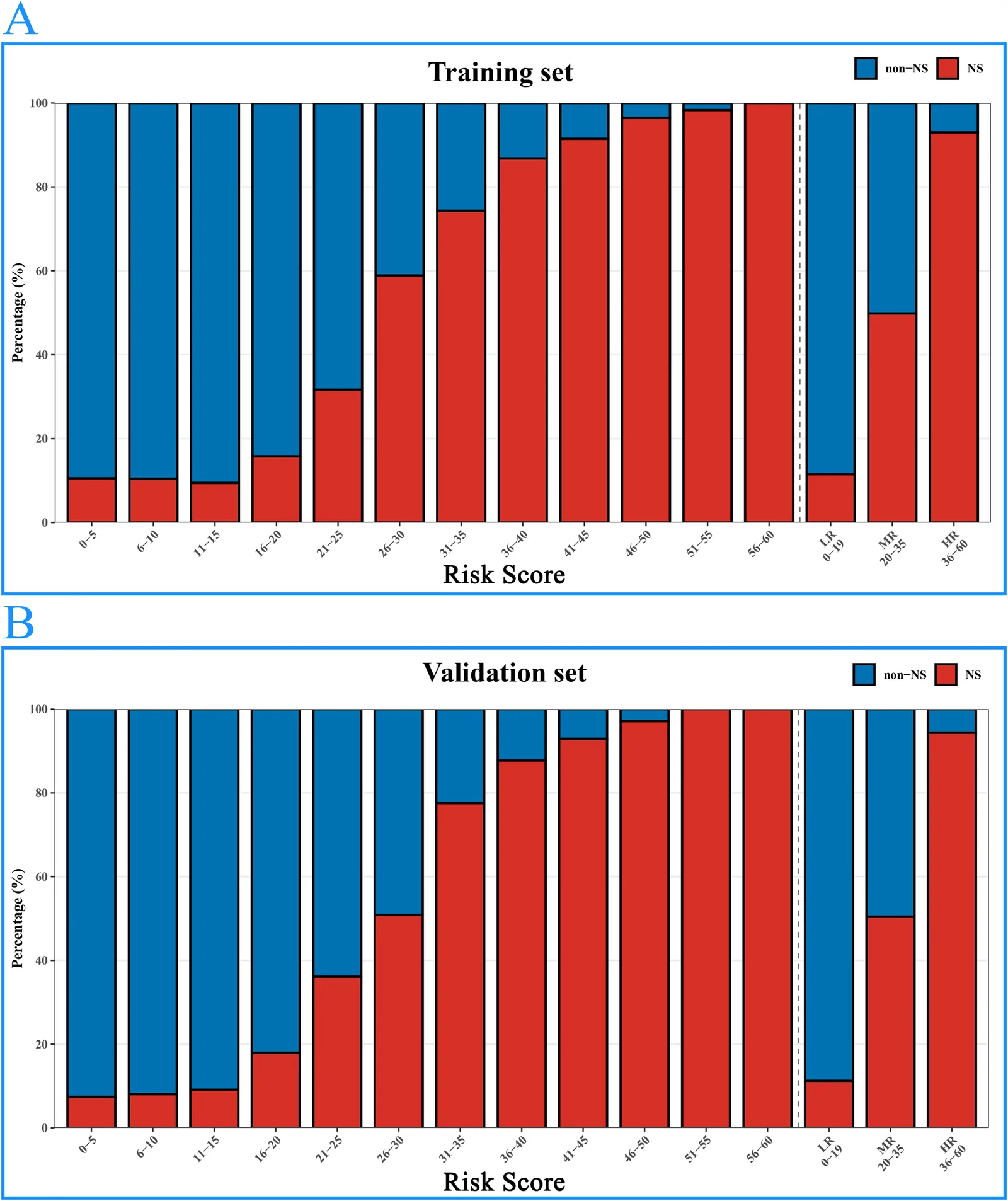
Distribution of patients across risk-score categories in the training and validation sets. **(A)** Percentage of patients in each risk-score category in the training set. **(B)** Percentage of patients in each risk-score category in the validation set. LR, low risk; MR, moderate risk; HR, high risk.

**Table 6:** Association between risk groups and NS diagnosis.

Variables	Training set					Internal validation set				
	β	S.E	Z	*P*	OR (95%CI)	β	S.E	Z	*P*	OR (95%CI)
Low risk [0, 19]					1.00 (Reference)					1.00 (Reference)
Moderate risk [20, 35]	2.03	0.08	25.18	<0.001	7.64 (6.52–8.95)	2.08	0.12	16.85	<0.001	8.03 (6.30–10.23)
High risk [36, 60]	4.63	0.11	41.88	<0.001	102.12 (82.24–126.80)	4.88	0.18	26.65	<0.001	132.15 (92.27–189.27)

NS, nephrotic syndrome; OR, odds ratio; CI, confidence interval.

## Discussion

In this study, we developed and internally validated a large-sample, multivariable diagnostic prediction model for adult NS. The model integrates 24 h UPE with a panel of routinely available clinical and laboratory indicators. To our knowledge, this is one of the largest single-center studies to date to develop a diagnostic prediction model for adult NS. The model showed good discrimination and calibration in the training and internal validation sets, but this performance was observed within the same single-center cohort. In patients with atypical clinical manifestations or borderline diagnostic results, the model may help integrate age, comorbidities, and laboratory results into diagnostic assessment. It is intended as an adjunct to clinical judgment in hospitalized adults with suspected NS and evidence of proteinuria.

### Current prediction models for NS

To date, numerous studies on prediction models for NS have been conducted worldwide. Yan et al. established a diagnostic model for minimal change disease by incorporating four indicators, namely diastolic blood pressure, serum immunoglobulin G, immunoglobulin M, and immunoglobulin E (14). Li et al. developed a model based on machine learning algorithms, which achieved a predictive accuracy of 76.1% for membranous nephropathy (19). Kou et al. developed an early-warning model for steroid-resistant NS in children that incorporated edema severity, 24 h UPE, serum albumin, cholesterol, and blood urea nitrogen (20). The prediction models developed by Wang et al. and Li et al. focused on the risk of clinical recurrence in patients with membranous nephropathy and minimal change NS after treatment, offering a quantitative tool for evaluating post-treatment recurrence risk (21, 22). Yang et al. also constructed an AKI prediction model specifically for patients with minimal change disease (23). Yu et al. built a model capable of predicting the risk of severe infection in children with idiopathic NS and developed an online risk calculator to enable rapid bedside assessment (24). Notably, no prediction model has been specifically developed for venous thromboembolism risk in NS patients to date, and the predictive efficacy of existing general internal medicine scoring systems remains limited in this patient population (6). In the present study, the significant predictive value of D-dimer may inform the future development of a disease-specific thrombosis risk prediction model for NS.

Existing models mainly focus on pathological classification, complications, recurrence, or prognosis and generally require a confirmed diagnosis of NS. This diagnostic model addresses the upstream diagnostic step in hospitalized adults with a preliminary diagnosis of NS and evidence of proteinuria. The score may help estimate the probability of NS and support diagnostic triage, particularly when clinical findings are atypical or borderline. It may complement, rather than replace, downstream pathological and prognostic models. However, treatment decisions and treatment outcomes were not evaluated in this study, and the model should not be used to select therapy or predict treatment response.

### Pathophysiological basis and clinical correlation of model variables

Within the diagnostic model, proteinuria-related indicators contributed most strongly to classification, with 24 h UPE ≥ 3.5 g/24 h carrying the greatest weight. This is not surprising: proteinuria is not merely a candidate biomarker but a core component of the KDIGO definition of NS, so its prominence reflects the model's alignment with the diagnostic standard it was designed to recapitulate. The model also retained sensitivity for presentations that fall outside a single proteinuria threshold, including patients with subnephrotic-range proteinuria who still satisfied the full diagnostic criteria, which may aid recognition of early or atypical glomerular injury without relying on any one cutoff. The link between proteinuria and NS is well established. Injury to the glomerular filtration barrier causes podocyte foot process effacement and loss of the charge barrier, leading to albumin leakage; proteinuria becomes massive once urinary loss exceeds the reabsorptive capacity of the renal tubules (25–27). Importantly, we explicitly acknowledge that 24 h UPE and serum albumin are components of the KDIGO diagnostic definition of NS, and their inclusion may therefore inflate the apparent discrimination of the full model. We interpret the model's high performance cautiously and position it as a diagnostic-assist tool rather than a replacement for established diagnostic criteria. The sensitivity analyses were designed to address this concern. After excluding 24 h UPE and qualitative urine protein, the reduced seven-variable model retained an AUC of 0.81 in both the training and internal validation sets, and after further excluding serum albumin, the six-variable model retained an AUC of 0.80 in both sets. The modest decline in AUC compared with the full model is expected and partly reflects overlap with the diagnostic definition, whereas the maintained performance of the reduced models indicates that the remaining clinical and laboratory predictors retain substantial independent diagnostic value. Subsequent hypoproteinemia induces a reduction in colloid osmotic pressure, which stimulates the renin-angiotensin axis and thereby causes sodium and fluid retention, ultimately leading to edema and hypertension (28). Meanwhile, hypoalbuminemia is a contributing factor to the compensatory hepatic synthesis of lipoproteins, which in turn triggers hyperlipidemia and the development of a hypercoagulable state in the body (29–34). Thromboembolic disease is frequently the initial presenting manifestation in patients with NS (3, 35); in the present study, the diagnostic value of a D-dimer ≥280 ng/mL underscores the unique role of the hypercoagulable state in NS, which facilitates the identification of patients at high risk of venous thromboembolism. The imbalance between increased procoagulant factors and depleted anticoagulant factors is the primary driver of hypercoagulability, and emerging evidence has confirmed that hyperlipidemia can promote thrombosis by enhancing platelet activity (33, 36, 37). Renal function markers such as serum creatinine in patients with NS are typically within the normal range at the initial clinical presentation, and these indices tend to rise as the disease progresses (32). Given that all enrolled patients were admitted to hospital with a preliminary diagnosis of proteinuria of unknown origin, renal function markers failed to show statistical significance in the present study.

In summary, the variables retained in the model are consistent with the pathophysiological network of NS. However, because the present study performed internal validation only, the model's external generalizability remains to be established in independent multicenter cohorts.

### Clinical implications for diagnostic support and risk stratification

In hospitalized adults with a preliminary diagnosis of NS and evidence of proteinuria, the model may support diagnostic assessment and risk stratification. To make the score clinically actionable, we defined three operational categories using tertiles of the total score in the training set: LR, 0–19 points; MR, 20–35 points; and HR, 36–60 points. In both sets, the proportion of patients with NS increased progressively across these categories, indicating that the score can stratify the diagnostic probability of NS. These categories are operational risk strata derived from score tertiles rather than diagnostic thresholds.

These categories are intended to guide diagnostic triage rather than treatment selection. For patients in the LR category, the probability of NS is lower, and evaluation for alternative causes with close follow-up may be appropriate, while invasive testing should not be performed solely on the basis of the score. For patients in the MR category, diagnostic uncertainty remains substantial, and nephrology evaluation with further diagnostic workup should be considered, including renal biopsy when clinically indicated. For patients in the HR category, the probability of NS is higher, supporting priority nephrology referral and expedited confirmatory evaluation, while the decision to perform renal biopsy should still follow standard clinical and pathological indications. The score is intended to support diagnostic assessment and risk communication.

The clinical relevance of these risk strata is further supported by the variables retained in the model. Proteinuria and hypertension are targets of RAAS blockade (36, 38), and SGLT_2_ inhibitors may further reduce proteinuria in selected patients after secondary causes have been excluded (39). Edema is commonly managed with diuretics (16, 29, 39), and albumin supplementation may improve diuretic response in severe hypoalbuminemia (36, 40). Hypoalbuminemia and a D-dimer level ≥280 ng/mL are associated with a prothrombotic state and may inform consideration of prophylactic anticoagulation (16, 36, 37, 41), while hyperlipidemia is managed with lipid-lowering therapy (36). These observations support the clinical relevance of the included variables and the rationale for the model. However, treatment outcomes were not evaluated in this study, and the model is not intended to guide treatment selection or predict treatment response.

### Strengths and limitations

The high prevalence of NS in adults and the associated increased risk of adverse outcomes impose a substantial disease burden on healthcare systems. However, specialized clinical diagnostic and assessment tools for adult NS remain limited. To our knowledge, this study provides one of the largest single-center evaluations of a diagnostic prediction model for adult NS using routinely available clinical and laboratory indicators. Nevertheless, several limitations should be acknowledged. First, although the model showed good discrimination and calibration in internal validation, its generalizability beyond this single-center cohort remains undetermined. External validation in independent multicenter populations is required. Second, compared with prospective study designs, the retrospective design confers a lower level of evidence and is subject to potential selection and information bias. Third, the model identifies the presence or absence of NS and cannot distinguish pathological subtypes because pathological data were not available for all enrolled cases. Fourth, 24 h UPE and serum albumin are components of the diagnostic criteria for NS, so the apparent discrimination of the full model is likely inflated. For high-risk patients in whom the clinical diagnosis remains uncertain, definitive diagnosis still depends on renal biopsy. Future research should integrate pathological data to develop subtype-specific models. The present findings require external validation before broader clinical use.

## Conclusion

This study reports a diagnostic prediction model for adult NS developed in a large single-center cohort and internally validated by bootstrap resampling. The model integrates routine clinical indicators, including age, proteinuria, hypoalbuminemia, edema, and D-dimer, and may support estimation of NS probability in the intended population. The AUC was 0.89 in the training set and 0.90 in the internal validation set, both derived from the same single-center cohort. The risk scoring system may improve clinical interpretability, but its broader clinical utility remains to be established. The model is currently intended for hospitalized patients with suspected NS and evidence of proteinuria. Future studies should assess its performance in outpatient and primary-care settings before broader application.

## Data Availability

The raw data supporting the conclusions of this article will be made available by the authors, without undue reservation.
