# Supplementary material for: Development and validation of a clinical prediction model for diagnosing nephrotic syndrome in adults: a retrospective diagnostic prediction study
Source: Front Med (Lausanne). 2026 Oct 21;13:1825840. doi: 10.3389/fmed.2026.1825840 (PMC13730878; doi:10.3389/fmed.2026.1825840)
Supplement: Supplementary file 1 [file Supplementaryfile1.docx]

**Supplemental materials**

**Figure S1.** Flow chart of the study screening.

**Table S1.** Comparison of observed and imputed values for variables with missing data in the training and internal validation sets.

**Figure S2.** LASSO regression.

**Table S2.** Dummy variable assignment table for each variable.

**Table S3.** Comparison between the training set and internal validation set before variable assignment.

**Table S4.** Comparison between the training set and internal validation set after variable assignment.

**Table S5.** Values of the GVIF for each variable.

**Table S6.** Sensitivity analyses after excluding proteinuria-related variables and serum albumin.

**Figure S3**. Sensitivity analysis of the NS diagnostic model.

**Table S7.** Proportion of patients with NS and non-NS in the training and internal validation sets at different score thresholds.

**Figure S1.** Flow chart of the screening of the study.

| **Table S1.** Comparison of observed and imputed values for variables with missing data in the training and internal validation sets | | | | | | | | | | |
| --- | --- | --- | --- | --- | --- | --- | --- | --- | --- | --- |
| Dataset | Variable | Nmissing | Observed  (mean) | Observed  (SD) | Imputed  (mean) | Imputed  (SD) | Mean difference | SMD | Absolute SMD | SD ratio |
| Training | 24h UPE | 260 | 2.3339 | 2.8462 | 2.9045 | 1.6251 | 0.5706 | 0.2462 | 0.2462 | 0.5710 |
| Training | Potassium | 131 | 3.7381 | 0.4990 | 3.7439 | 0.0731 | 0.0059 | 0.0165 | 0.0165 | 0.1465 |
| Training | Sodium | 133 | 139.2017 | 3.8653 | 139.2134 | 0.6286 | 0.0117 | 0.0042 | 0.0042 | 0.1626 |
| Training | Serum creatinine | 244 | 102.9197 | 114.2867 | 107.4314 | 25.4235 | 4.5116 | 0.0545 | 0.0545 | 0.2225 |
| Training | Uric acid | 156 | 350.9807 | 109.1647 | 357.2620 | 33.2581 | 6.2813 | 0.0778 | 0.0778 | 0.3047 |
| Training | Urea | 156 | 7.3294 | 4.8309 | 7.5412 | 1.4329 | 0.2118 | 0.0594 | 0.0594 | 0.2966 |
| Training | Serum albumin | 109 | 29.5785 | 9.7100 | 29.6567 | 3.2235 | 0.0783 | 0.0108 | 0.0108 | 0.3320 |
| Training | Magnesium | 311 | 0.8405 | 0.1051 | 0.8444 | 0.0149 | 0.0040 | 0.0529 | 0.0529 | 0.1418 |
| Training | Cystatin C | 62 | 1.3115 | 0.8202 | 1.3360 | 0.1991 | 0.0246 | 0.0411 | 0.0411 | 0.2427 |
| Training | D-dimer | 1143 | 476.8571 | 951.9912 | 439.7268 | 235.5771 | -37.1303 | -0.0535 | 0.0535 | 0.2475 |
| Training | Procalcitonin | 60 | 0.3727 | 3.0605 | 0.4230 | 0.1673 | 0.0503 | 0.0232 | 0.0232 | 0.0547 |
| Training | Interleukin-6 | 53 | 20.1454 | 115.7822 | 19.2732 | 6.5201 | -0.8722 | -0.0106 | 0.0106 | 0.0563 |
| Validation | 24h UPE | 103 | 2.3632 | 2.8627 | 2.9355 | 1.7740 | 0.5723 | 0.2403 | 0.2403 | 0.6197 |
| Validation | Potassium | 42 | 3.7410 | 0.5047 | 3.7305 | 0.0994 | -0.0104 | -0.0287 | 0.0287 | 0.1970 |
| Validation | Sodium | 42 | 139.2448 | 3.8652 | 138.9974 | 0.7812 | -0.2474 | -0.0887 | 0.0887 | 0.2021 |
| Validation | Serum creatinine | 82 | 101.1821 | 107.5288 | 102.6916 | 24.7550 | 1.5096 | 0.0193 | 0.0193 | 0.2302 |
| Validation | Uric acid | 55 | 349.4820 | 105.6825 | 351.7569 | 31.2286 | 2.2749 | 0.0292 | 0.0292 | 0.2955 |
| Validation | Urea | 55 | 7.2374 | 4.5443 | 7.4235 | 1.4467 | 0.1861 | 0.0552 | 0.0552 | 0.3183 |
| Validation | Serum albumin | 31 | 29.0834 | 9.8234 | 27.9567 | 3.0714 | -1.1267 | -0.1548 | 0.1548 | 0.3127 |
| Validation | Magnesium | 110 | 0.8385 | 0.1048 | 0.8420 | 0.0121 | 0.0036 | 0.0476 | 0.0476 | 0.1158 |
| Validation | Cystatin C | 25 | 1.2912 | 0.7826 | 1.2375 | 0.1542 | -0.0537 | -0.0953 | 0.0953 | 0.1971 |
| Validation | D-dimer | 492 | 476.3800 | 1160.9787 | 435.9288 | 243.0441 | -40.4512 | -0.0482 | 0.0482 | 0.2093 |
| Validation | Procalcitonin | 31 | 0.3688 | 3.4095 | 0.4322 | 0.2471 | 0.0633 | 0.0262 | 0.0262 | 0.0725 |
| Validation | Interleukin-6 | 23 | 17.9936 | 81.1084 | 17.2040 | 5.0637 | -0.7896 | -0.0137 | 0.0137 | 0.0624 |

**Notes.** 24h UPE, 24-hour urinary protein excretion. SD, standard deviation. SMD, standardized mean difference.

**Figure S2.** LASSO regression. (A) LASSO regression variable trajectory plot; (B) LASSO regression variable selection flowchart.

**Table S2**. Dummy variable assignment table for each variable

| Variables | Rules for assigning dummy variables |
| --- | --- |
| Event | non-NS = 0, NS = 1 |
| Hypertension | No = 0, Yes = 1 |
| Hyperlipidemia | No = 0, Yes = 1 |
| Edema | No = 0, Yes = 1 |
| Urine protein qualitative | Negative = 0, 1+ = 1, 2+ = 2, 3+ = 3, 4+ = 4 |
| Age, years | ≥65 = 0, [45, 64] = 1, [18, 44] = 2 |
| 24h UPE, g/24h | [0,0.15) = 0,[0.15,3.5) = 1,≥3.5 = 2 |
| Serum albumin, g/L | [39, 54] = 0, <39 = 1, >54 = 2 |
| D-dimer, ng/mL | [0, 280) = 0, ≥280 = 1 |
| Serum creatinine, μmol/L | [33, 75] = 0, <33 = 1, >75 = 2 |
| Cystatin C, mg/L | [0.56, 1.15] = 0, <0.56 = 1, >1.15 = 2 |

**Notes**. 24h UPE: 24-hour urinary protein excretion; All quantitative laboratory variables were categorized according to the reference values provided by the clinical laboratory of our hospital. These thresholds were defined a priori before model development and were not derived from data-driven threshold optimization.

**Table S3.** Comparison between the training set and internal validation set before variable assignment

| Variables | Total  (n = 8950) | Internal validation set  (n = 2685) | Training set  (n = 6265) | Z/χ^2^ | P |
| --- | --- | --- | --- | --- | --- |
| Age, years | 51.00  (38.00, 62.00) | 51.00  (38.00, 61.00) | 51.00  (38.00, 62.00) | 0.65 | 0.515 |
| 24h UPE, g/24h | 1.39  (0.19, 4.02) | 1.38  (0.18, 4.03) | 1.40  (0.19, 4.02) | 0.1 | 0.923 |
| Alb, g/L | 28.50  (21.87, 37.00) | 28.00  (21.40, 36.80) | 28.70  (22.10, 37.10) | 2.54 | 0.011 |
| Scr, μmol/L | 77.24  (60.59,103.71) | 77.00  (60.00, 102.00) | 77.50  (60.94, 104.46) | 0.98 | 0.325 |
| Cys C, mg/L | 1.09  (0.85, 1.47) | 1.09  (0.85, 1.45) | 1.09  (0.85, 1.47) | 0.74 | 0.462 |
| D-dimer, ng/mL | 235.74  (109.00,510.00) | 229.58  (111.00, 500.00) | 238.00  (109.00, 515.00) | 0.49 | 0.627 |
| Event, |  |  |  | 0 | 1 |
| non-NS | 4400 (49.16) | 1320 (49.16) | 3080 (49.16) |  |  |
| NS | 4550 (50.84) | 1365 (50.84) | 3185 (50.84) |  |  |
| Hypertension, |  |  |  | 1.8 | 0.18 |
| No | 4225 (47.21) | 1297 (48.31) | 2928 (46.74) |  |  |
| Yes | 4725 (52.79) | 1388 (51.69) | 3337 (53.26) |  |  |
| Hyperlipidemia, |  |  |  | 0.31 | 0.578 |
| No | 7230 (80.78) | 2179 (81.15) | 5051 (80.62) |  |  |
| Yes | 1720 (19.22) | 506 (18.85) | 1214 (19.38) |  |  |
| Edema, |  |  |  | 0.2 | 0.656 |
| No | 4047 (45.22) | 1204 (44.84) | 2843 (45.38) |  |  |
| Yes | 4903 (54.78) | 1481 (55.16) | 3422 (54.62) |  |  |
| Urine protein qualitative |  |  |  | 2.58 | 0.63 |
| Negative | 1789 (19.99) | 541 (20.15) | 1248 (19.92) |  |  |
| 1+ | 1563 (17.46) | 466 (17.36) | 1097 (17.51) |  |  |
| 2+ | 1740 (19.44) | 542 (20.19) | 1198 (19.12) |  |  |
| 3+ | 3670 (41.01) | 1075 (40.04) | 2595 (41.42) |  |  |
| 4+ | 188 (2.10) | 61 (2.27) | 127 (2.03) |  |  |

**Notes.** Continuous variables are presented as median (Q1, Q3), and categorical variables as n (%). Comparisons between the training and internal validation sets were performed using the Mann-Whitney U test for continuous variables and the chi-square test for categorical variables. Z, Mann-Whitney U test statistic. χ², chi-square test statistic. M, median. Q_1_, first quartile. Q_3_, third quartile. 24h UPE, 24-hour urinary protein excretion. Alb, serum albumin. Scr, serum creatinine. CysC, cystatin C. NS, nephrotic syndrome.

**Table S4**. Comparison between the training set and internal validation set after variable assignment

| Variables | Total  (n = 8950) | Internal validation set  (n = 2685) | Training set  (n = 6265) | χ² | P |
| --- | --- | --- | --- | --- | --- |
| Event, n (%) |  |  |  | 0 | 1 |
| non-NS | 4400 (49.16) | 1320 (49.16) | 3080 (49.16) |  |  |
| NS | 4550 (50.84) | 1365 (50.84) | 3185 (50.84) |  |  |
| Hypertension, n (%) |  |  |  | 1.8 | 0.18 |
| No | 4225 (47.21) | 1297 (48.31) | 2928 (46.74) |  |  |
| Yes | 4725 (52.79) | 1388 (51.69) | 3337 (53.26) |  |  |
| Hyperlipidemia, n (%) |  |  |  | 0.31 | 0.578 |
| No | 7230 (80.78) | 2179 (81.15) | 5051 (80.62) |  |  |
| Yes | 1720 (19.22) | 506 (18.85) | 1214 (19.38) |  |  |
| Edema, n (%) |  |  |  | 0.2 | 0.656 |
| No | 4047 (45.22) | 1204 (44.84) | 2843 (45.38) |  |  |
| Yes | 4903 (54.78) | 1481 (55.16) | 3422 (54.62) |  |  |
| 24h UPE, n (%) |  |  |  | 1.09 | 0.579 |
| <0.15 | 2007 (22.42) | 621 (23.13) | 1386 (22.12) |  |  |
| 0.15-3.499 | 4219 (47.14) | 1254 (46.70) | 2965 (47.33) |  |  |
| ≥3.5 | 2724 (30.44) | 810 (30.17) | 1914 (30.55) |  |  |
| Urine protein qualitative, n (%) |  |  |  | 2.58 | 0.63 |
| Negative | 1789 (19.99) | 541 (20.15) | 1248 (19.92) |  |  |
| 1+ | 1563 (17.46) | 466 (17.36) | 1097 (17.51) |  |  |
| 2+ | 1740 (19.44) | 542 (20.19) | 1198 (19.12) |  |  |
| 3+ | 3670 (41.01) | 1075 (40.04) | 2595 (41.42) |  |  |
| 4+ | 188 (2.10) | 61 (2.27) | 127 (2.03) |  |  |
| Age (years), n (%) |  |  |  | 1 | 0.607 |
| ≥65 | 1845 (20.61) | 539 (20.07) | 1306 (20.85) |  |  |
| 45-64 | 3916 (43.75) | 1172 (43.65) | 2744 (43.80) |  |  |
| 18-44 | 3189 (35.63) | 974 (36.28) | 2215 (35.36) |  |  |
| Serum albumin, n (%) |  |  |  | 0.71 | 0.7 |
| 39-54 | 1664 (18.59) | 485 (18.06) | 1179 (18.82) |  |  |
| <39 | 7214 (80.60) | 2178 (81.12) | 5036 (80.38) |  |  |
| >54 | 72 (0.80) | 22 (0.82) | 50 (0.80) |  |  |
| Serum creatinine, n (%) |  |  |  | 1.25 | 0.537 |
| 33-75 | 4160 (46.48) | 1262 (47.00) | 2898 (46.26) |  |  |
| <33 | 63 (0.70) | 22 (0.82) | 41 (0.65) |  |  |
| >75 | 4727 (52.82) | 1401 (52.18) | 3326 (53.09) |  |  |
| Cystatin C, n (%) |  |  |  | 2.32 | 0.314 |
| 0.56-1.15 | 4772 (53.32) | 1431 (53.30) | 3341 (53.33) |  |  |
| <0.56 | 229 (2.56) | 79 (2.94) | 150 (2.39) |  |  |
| >1.15 | 3949 (44.12) | 1175 (43.76) | 2774 (44.28) |  |  |
| D-dimer, n (%) |  |  |  | 0.34 | 0.559 |
| <280 | 4973 (55.56) | 1505 (56.05) | 3468 (55.36) |  |  |
| ≥280 | 3977 (44.44) | 1180 (43.95) | 2797 (44.64) | 0.34 | 0.559 |

**Notes.** χ², chi-square test statistic. 24h UPE, 24-hour urinary protein excretion. NS, nephrotic syndrome. Scr, serum creatinine. CysC, cystatin C.

**Table S5.** Values of the GVIF for each variable

| Variables | GVIF | Df | GVIF^(1/(2*Df)) |
| --- | --- | --- | --- |
| Age, years | 1.257 | 2 | 1.059 |
| Hypertension | 1.143 | 1 | 1.069 |
| Hyperlipidemia | 1.072 | 1 | 1.035 |
| Edema | 1.287 | 1 | 1.134 |
| 24h UPE | 2.641 | 2 | 1.275 |
| Urine protein qualitative | 2.611 | 4 | 1.127 |
| Serum albumin | 1.114 | 2 | 1.027 |
| D-dimer | 1.302 | 1 | 1.141 |
| Cystatin C | 1.127 | 2 | 1.030 |

**Notes.** GVIF: Generalized Variance Inflation Factor; 24h UPE, 24-hour urinary protein excretion

**Table S6.** Sensitivity analyses after excluding proteinuria-related variables and serum albumin.

| Sensitivity analysis | Data | Cutoff | AUC (95%CI) | Accuracy  (95%CI) | Sensitivity  (95%CI) | Specificity  (95%CI) | PPV  (95%CI) | NPV  (95%CI) |
| --- | --- | --- | --- | --- | --- | --- | --- | --- |
| Model 1 | Training set | 0.54 | 0.81 (0.80-0.83) | 0.74 (0.73-0.76) | 0.73 (0.72-0.75) | 0.76 (0.74-0.77) | 0.76 (0.74-0.77) | 0.73 (0.72-0.75) |
|  | internal validation set | 0.54 | 0.81 (0.80-0.83) | 0.74 (0.72-0.75) | 0.74 (0.72-0.77) | 0.74 (0.71-0.76) | 0.74 (0.72-0.77) | 0.73 (0.71-0.76) |
| Model 2 | Training set | 0.55 | 0.80 (0.79-0.81) | 0.73 (0.72-0.74) | 0.70 (0.69-0.72) | 0.76 (0.75-0.78) | 0.75 (0.74-0.77) | 0.71 (0.70-0.73) |
|  | internal validation set | 0.55 | 0.80 (0.78-0.81) | 0.73 (0.71-0.75) | 0.71 (0.69-0.73) | 0.75 (0.73-0.77) | 0.75 (0.72-0.77) | 0.71 (0.69-0.74) |

**Notes.** Model 1 excluded 24h UPE and urine protein qualitative. Model 2 further excluded serum albumin. AUC, area under the receiver operating characteristic curve. CI, confidence interval. PPV, positive predictive value. NPV, negative predictive value.

**Figure S3. Sensitivity analysis of the NS diagnostic model.**

**Notes.** (A) ROC curves after excluding 24h UPE and urine protein qualitative. (B) ROC curves after further excluding serum albumin.

**Table S7.** Distribution of patients with NS and non-NS by total score interval and risk group in the training and internal validation sets

| **Score** | **Training set** | | | **Internal validation set** | | |
| --- | --- | --- | --- | --- | --- | --- |
|  | **n** | **non-NS, n (%)** | **NS, n (%)** | **n** | **non-NS, n (%)** | **NS, n (%)** |
| 0-5 | 114 | 102 (89.47) | 12 (10.53) | 54 | 50 (92.59) | 4 (7.41) |
| 6-10 | 566 | 507 (89.58) | 59 (10.42) | 236 | 217 (91.95) | 19 (8.05) |
| 11-15 | 710 | 643 (90.56) | 67 (9.44) | 307 | 279 (90.88) | 28 (9.12) |
| 16-20 | 932 | 785 (84.23) | 147 (15.77) | 385 | 316 (82.08) | 69 (17.92) |
| 21-25 | 711 | 486 (68.35) | 225 (31.65) | 335 | 214 (63.88) | 121 (36.12) |
| 26-30 | 683 | 281 (41.14) | 402 (58.86) | 289 | 142 (49.13) | 147 (50.87) |
| 31-35 | 521 | 134 (25.72) | 387 (74.28) | 245 | 55 (22.45) | 190 (77.55) |
| 36-40 | 424 | 56 (13.21) | 368 (86.79) | 155 | 19 (12.26) | 136 (87.74) |
| 41-45 | 680 | 58 (8.53) | 622 (91.47) | 267 | 19 (7.12) | 248 (92.88) |
| 46-50 | 675 | 24 (3.56) | 651 (96.44) | 312 | 9 (2.88) | 303 (97.12) |
| 51-55 | 236 | 4 (1.69) | 232 (98.31) | 95 | 0 (0.00) | 95 (100.00) |
| 56-60 | 13 | 0 (0.00) | 13 (100.00) | 5 | 0 (0.00) | 5 (100.00) |
| LR [0,19] | 2120 | 1876 (88.49) | 244 (11.51) | 907 | 805 (88.75) | 102 (11.25) |
| MR [20,35] | 2117 | 1062 (50.17) | 1055 (49.83) | 944 | 468 (49.58) | 476 (50.42) |
| HR [36,60] | 2028 | 142 (7.00) | 1886 (93.00) | 834 | 47 (5.64) | 787 (94.36) |

**Notes.** L:low risk, M:moderate risk, H:high risk.
